# Effects of a zoonotic pathogen, *Borrelia burgdorferi*, on the behavior of a key reservoir host

**DOI:** 10.1002/ece3.3961

**Published:** 2018-03-26

**Authors:** Richard S. Ostfeld, Dustin Brisson, Kelly Oggenfuss, Jill Devine, Michael Z. Levy, Felicia Keesing

**Affiliations:** ^1^ Cary Institute of Ecosystem Studies Millbrook NY USA; ^2^ University of Pennsylvania Philadelphia PA USA; ^3^ Bard College Annandale‐on‐Hudson NY USA

**Keywords:** Lyme disease, reservoir host, tick‐borne disease, vaccination, wildlife reservoir, zoonosis, zoonotic disease

## Abstract

Most emerging infectious diseases of humans are transmitted to humans from other animals. The transmission of these “zoonotic” pathogens is affected by the abundance and behavior of their wildlife hosts. However, the effects of infection with zoonotic pathogens on behavior of wildlife hosts, particularly those that might propagate through ecological communities, are not well understood. *Borrelia burgdorferi* is a bacterium that causes Lyme disease, the most common vector‐borne disease in the USA and Europe. In its North American range, the pathogen is most frequently transmitted among hosts through the bite of infected blacklegged ticks (*Ixodes scapularis*). Using sham and true vaccines, we experimentally manipulated infection load with this zoonotic pathogen in its most competent wildlife reservoir host, the white‐footed mouse, *Peromyscus leucopus*, and quantified the effects of infection on mouse foraging behavior, as well as levels of mouse infestation with ticks. Mice treated with the true vaccine had 20% fewer larval blacklegged ticks infesting them compared to mice treated with the sham vaccine, a significant difference. We observed a nonsignificant trend for mice treated with the true vaccine to be more likely to visit experimental foraging trays (20%–30% effect size) and to prey on gypsy moth pupae (5%–20% effect size) compared to mice treated with the sham vaccine. We observed no difference between mice on true‐ versus sham‐vaccinated grids in risk‐averse foraging. Infection with this zoonotic pathogen appears to elicit behavioral changes that might reduce self‐grooming, but other behaviors were affected subtly or not at all. High titers of *B. burgdorferi* in mice could elicit a self‐reinforcing feedback loop in which reduced grooming increases tick burdens and hence exposure to tick‐borne pathogens.

## 1 INTRODUCTION

The majority of emerging infectious diseases of humans are transmitted to humans from other vertebrates; that is, they are “zoonotic” (Taylor, Latham, & Woolhouse, 2001; Woolhouse & Gowtage‐Sequeria, 2005). Zoonotic pathogens typically infect one or more wildlife host species in addition to humans. Rarely, however, have the effects of zoonotic pathogens on their wildlife hosts, including behaviors, been considered (Gage and Kosoy 2005, Luis et al. 2012, George et al. 2013, Worth et al. 2014), and we know of no studies examining the impacts of zoonotic pathogens on interactions between reservoir hosts and other members of the ecological communities of which they are a part. The effects of a zoonotic pathogen on host behavior, population dynamics, or competitive interactions could have significant ecological consequences that radiate through an ecosystem. For instance, zoonotic pathogens typically infect multiple host species, but some hosts amplify pathogen populations and others do not (Ostfeld & Keesing, 2012). Transmission of the pathogen from infected hosts to new hosts depends critically on the population density and behavior of the infected hosts. Both of these factors could be affected by the pathogen. Some host species also play other important roles in ecosystems—for example as keystone or hub species—and their roles in communities could be affected by changes in behavior or abundance caused by the pathogen (Collinge & Ray, 2007; Dobson & Hudson, 1986).

We examined the effects of a widespread zoonotic pathogen, the Lyme disease bacterium *Borrelia burgdorferi* (hereafter *Bb*), on a key reservoir host, the white‐footed mouse (*Peromyscus leucopus*) (Brisson, Dykhuizen, & Ostfeld, 2008; LoGiudice, Ostfeld, Schmidt, & Keesing, 2003). Lyme disease is the most prevalent vector‐borne disease in the temperate zone worldwide, and its incidence rates and geographic extent are both expanding rapidly (Kugeler, Farley, Forrester, & Mead, 2015; Ostfeld, 2011). The pathogen is transmitted to vertebrate hosts through bites by ixodid ticks, which serve as its vectors (Ostfeld, 2011). In eastern North America, the vector species is the blacklegged tick (*Ixodes scapularis*). Larvae of these ticks hatch uninfected with the Lyme disease bacterium, but they can acquire the bacterium from a host during their larval blood meal. If the ticks acquire *Bb* infection during their larval meal, they can then transmit the infection to various hosts, including humans, during the nymphal or adult blood meal (Brisson, Drecktrah, Eggers, & Samuels, 2012; LoGiudice et al., 2003).

The consequences for the host of becoming infected with *Bb* vary from species to species. In humans, *Bb* infections can produce arthritis, carditis, facial palsy, chronic fatigue, and other symptoms (Cairns & Godwin, 2005; Massarotti, 2002; Pinto, 2002; Rahn, 1991). *Bb* also causes serious disease in dogs as a result of kidney, synovial, and nervous system lesions (Day, 2011) and meningitis and neuritis in horses (James, Engiles, & Beech, 2010). Some strains of laboratory mice (*Mus musculus*) also experience damaged skeletomuscular and neurological tissues from *Bb* infection (Barthold, 1991; Burgess, French, & Gendron‐Fitzpatrick, 1990; Moro et al., 2002).

*Peromyscus leucopus* is the most competent reservoir host for *Bb*, with some studies demonstrating that mice transmit infection to ≥80% of feeding larval ticks (Brisson & Dykhuizen, 2004; LoGiudice et al., 2003). The effects of *Bb* infection on white‐footed mice have not been clearly determined. Burgess et al. (1990) described neurological disease and lesions in brain, kidney, and liver of spirochete‐infected mice, but the study was correlative and thus did not establish *Bb* infection as the cause of these symptoms. In contrast, others (Schwanz, Voordouw, Brisson, & Ostfeld, 2011) found no difference between *Bb*‐infected and uninfected mice in either innate immune response or wheel‐running activity in the laboratory. Further, Hofmeister, Ellis, Glass, and Childs (1999) compared survival times of mice that became infected during their study (*N* = 28, median survival = 123 days) and those that did not (*N* = 19, median survival = 97 days). Despite the apparently longer survival of infected mice, a comparison of medians with a Wilcoxon test showed this difference to be nonsignificant (Hofmeister et al., 1999), although sample sizes were small and the tests were conservative.

The effects of *Bb* infection on *P. leucopus* are particularly important ecologically. These mice are a critical hub species in eastern forests. Mice serve as prey for raptors and carnivores (Levi, Kilpatrick, Mangel, & Wilmers, 2012; Schmidt & Ostfeld, 2008) and eat the seeds of forest trees (Manson, Ostfeld, & Canham, 2001; Schnurr, Ostfeld, & Canham, 2002), the eggs of ground‐nesting songbirds (Schmidt et al., 2001), and the pupae of gypsy moths (Elkinton et al., 1996; Jones, Ostfeld, Richard, Schauber, & Wolff, 1998). For example, mice attack eggs in the nests of veeries (*Catharus fuscescens*), causing nest failure when mice are at high density (Schmidt & Ostfeld, 2008). The effects of this predation are pronounced enough that regional declines in veery abundance can be detected in Breeding Bird Survey data when mouse populations have been high (Schmidt, 2003). Mice are also important predators on the pupal stage of gypsy moths, an invasive forest pest that can defoliate forests when at peak density (Elkinton et al., 1996; Ostfeld, Jones, & Wolff, 1996). The moths pupate on the trunks of trees, which leaves them vulnerable to predation by mice during a several‐week window in midsummer. When mice are at high densities, virtually all of the pupae can be killed; when mice are at low densities, large numbers of pupae survive, setting the stage for a moth outbreak (Jones et al., 1998).

To determine the effects of *Bb* on ecological interactions in forests, we experimentally manipulated *Bb* infection loads in mice using an injectable anti‐*Bb* vaccine patterned after Tsao et al. (2004) as well as a control vaccine. Using these vaccines, we assessed the effects of *Bb* infection load on movement and foraging behavior by mice. We assessed foraging behavior directly by quantifying the impact of the vaccination status of mice (vaccinated or sham‐vaccinated) on experimentally deployed pupae of gypsy moths. We also measured foraging behavior indirectly by focusing on whether *Bb* infection load affected risk aversion by mice. This is a key behavior affecting interactions between mice and their prey—gypsy moths, ground‐nesting songbirds, and tree seeds (Schmidt & Ostfeld, 2008; Schwanz, Previtali, Gomes‐Solecki, Brisson, & Ostfeld, 2012). We also assessed whether *Bb* infection load affected numbers of larval blacklegged ticks infesting mouse hosts. If high *Bb* infection load reduces the total amount of space used by individual mice, we expected mice on the anti‐*Bb‐*vaccinated plots to have greater tick burdens, owing to higher encounter rates. If high *Bb* infection load reduces the intensity of self‐grooming behavior, we expected mice on the anti‐*Bb*‐vaccinated plots to have lower tick burdens.

## 2 METHODS

### 2.1 Development of true and sham vaccines

We developed an anti‐*Bb* “true” vaccine patterned after the *Bb* OspA vaccine used by Tsao et al. (2004). We also developed a sham vaccine based on the OspA protein of the congener *B. garinii* (hereafter *Bg*) to administer to mice as a control for the immunochallenge of receiving a vaccine (Schwanz et al., 2012). For the true vaccine, we cloned OspA from *Bb* strain B31 into the protein expression vector pET45b creating an OspA‐6XHis fusion that was transformed into *Escherichia coli* BL21. For the sham vaccine, we cloned and transformed the full‐length OspA from *Bg* strain LV4. Production and purification were as previously described (Voordouw et al., 2013), and purity was assessed by SDS‐PAGE and mass spectrometry.

### 2.2 Capturing and immunizing animals

We conducted our field studies on six 2.25‐ha forested plots at the Cary Institute of Ecosystem Studies in Millbrook, NY, USA. These plots, which have been trapped continuously since 1995, are in oak‐dominated mixed deciduous forest. The six plots are arranged as three sets of pairs based on physical proximity, with the plots within each pair separated by 150 to 200 m, and the pairs separated by 500 to 2,500 m. Five of the six plots contain an 11 × 11 point grid, and the sixth plot has a 10 × 12 point grid, of Sherman live traps, with two traps at each grid point and 15 m between trap stations. Between April and November 2015, we conducted trapping every 2–3 weeks for two consecutive nights on each grid. Traps were baited with crimped oats, with sunflower seeds added when temperatures were below 10°C. We set traps at 16:00 each afternoon and checked them the following morning between 08:00 and 11:00. Capture probabilities for mice per two‐day trap session typically exceed 0.85. Captured small mammals were marked with individually numbered ear tags if not previously marked and were then characterized for sex, age, mass, and reproductive maturity. We also counted the number of ticks on the head and ears so that we could compare the tick burdens of mice on sham‐vaccinated versus true‐vaccinated grids. We estimated population density on each plot using the minimum number alive (MNA) method (Slade & Blair, 2000), which sums for each two‐day trapping session, the number of individuals caught in a trapping session plus those caught both before and after (but not during) that specific trapping session.

Each grid also contained 40 evenly spaced wooden nest boxes attached to tree trunks at chest height. We supplied these boxes, which are often used by adult females for raising litters from birth to weaning, with cotton nesting materials and checked them every 2–4 weeks. Because gestation in white‐footed mice is ~28 days, this schedule allowed us to detect a high proportion of litters born on each grid.

From April through September 2015, we immunized mice weighing more than 10 g and captured either in the nest boxes or in traps with either the true or the sham vaccine. Mice living on three of the grids, hereafter “experimental” grids, were given the true vaccine, which protects against *B. burgdorferi*. Mice living on the remaining three grids, hereafter “control” grids, were given the sham vaccine against *B. garinii*, which served as a control for the immunochallenge. Following Tsao et al. (2004), we subcutaneously inoculated mice with 10 μg of either true vaccine (*Bb* OspA) or sham vaccine (*Bg* OspA) suspended in a 1:1 ratio of Freund's adjuvant (Sigma‐Aldrich) upon first capture, with that suspension made fresh each day. We gave recaptured mice up to two 10 μg boosters during subsequent trapping sessions, with each shot occurring at least 12 days after the previous one. To assess the anti‐OspA response by ELISA, we took blood from the submandibular vein of each animal before the administration of the first vaccine and then after the administration of the second booster, which was a minimum of 24 days later. At the time of blood sampling, we also took 2 mm ear‐punch biopsies of each animal. Tissue was taken on the same schedule as the blood sample, immediately before the first dose and immediately following the second booster. After handling, animals were released at the point of capture.

### 2.3 Determining infection status of mice

Infection status and reservoir competence of mice were determined using xenodiagnosis. We retrieved a sample of 10–15 true‐vaccinated and 10–15 sham‐vaccinated mice per plot to the Animal Rearing Facility at the Cary Institute of Ecosystem Studies in August 2015, which is the period of peak larval tick activity (Levi, Keesing, Oggenfuss, & Ostfeld, 2015). For 3–5 days, which is the duration of feeding for larval ticks, we housed the mice in wire‐mesh cages suspended over pans lined with moistened paper towels and edged with a barrier of petroleum jelly. Approximately 30 larval ticks were added to the head and ears of any mouse that had fewer than 10 ticks from the field following methods described in Keesing et al. (2009). Briefly, we collected larval ticks at locations off of our grids, and then added them to the skin of each mouse with a paintbrush. We held mice immobile for 2 hr in an aerated PVC tube, and then returned them to their cages and gave them food and water ad libitum. Mice with added ticks were kept for 5 days, while those with naturally acquired tick burdens were kept for 3 days. We collected all replete larval ticks that fell into the pans, allowed them to molt to the nymphal stage (approximately 1 month) following procedures described in detail in (Hersh et al., 2014), and tested the resulting nymphs by PCR for infection with *Bb* (Brisson & Dykhuizen, 2004; Brisson et al., 2008). *Bb* DNA was amplified by nested PCR of the *ospC* locus as previously described (Brisson & Dykhuizen, 2004) and the *rrs‐rrlA* intergenic spacer (IGS) locus (Bunikis et al., 2004).

### 2.4 Measuring foraging behavior

**Figure 1:**
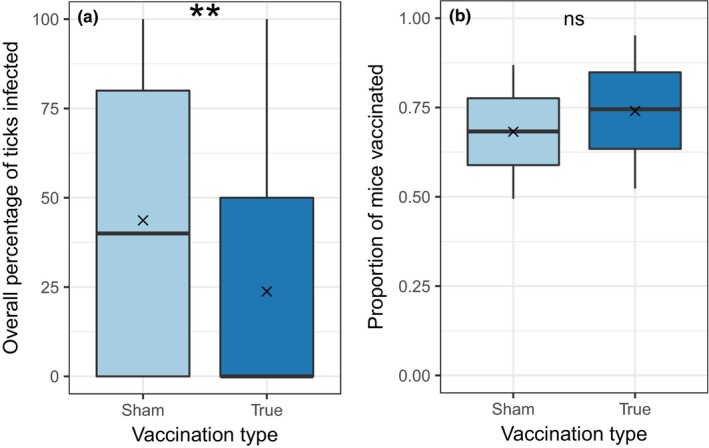
(a) Efficacy of the vaccine. Mice vaccinated with the sham vaccine were twice as likely to infect feeding larval ticks as were mice vaccinated with the true vaccine (Poisson model with an offset of the total ticks tested; *p* = .00026). (b) The proportion of mice on the grids that had been vaccinated with at least one dose of either the sham or the true vaccine at the start of experiments with seed trays and gypsy moth pupae were not different. × denotes the mean value. **p < .01; ns, not significant

To measure risk aversion, we determined the quitting harvest rate (QHR) of mice on the field plots, defined as the rate of food acquisition at which a forager leaves a patch. The QHR can be estimated and compared among patches by measuring the time a forager spends in a patch, or the giving‐up density (GUD) of a food patch, which is the amount of food (e.g., seed) left behind when a forager leaves a food patch (Brown, 1988, 1992). Food patches with higher costs of predation (i.e., riskier habitats) have been shown to have higher GUDs, indicating that foragers have a higher QHR in these patches and thus require greater food intake rates to remain foraging in a risky habitat (Brown, 1988; Brown & Kotler, 2004). In previous research, we measured foraging behavior of mice on our trapping grids using giving‐up densities of seeds at experimental seed trays that were either risky (uncovered) or safe (covered) patches (Schwanz, Brisson, Gomes‐Solecki, & Ostfeld, 2011). The difference in the amount of seed removed from uncovered trays versus covered trays provides an index of risk sensitivity. When mice show much greater preference for foraging in covered trays, they are highly sensitive to risks; when they show a smaller difference, they show lower risk sensitivity. Mice with lower risk sensitivity are more likely to forage across a wide range of microhabitats, decreasing “mouse‐free space” (Schauber, Goodwin, Jones, & Ostfeld, 2007; Schmidt, 2004). To measure the GUD of individual mice that varied in infection status, we deployed foraging arenas on nontrapping nights in June and July 2015, after an average of ~70% of mice had been either true‐vaccinated or sham‐vaccinated (Figure 1b). Following the methods of Schwanz, Voordouw, et al. (2011), Schwanz, Brisson, et al. (2011) and Schwanz et al. (2012), we placed two arenas within 2 m of a trap site. The arenas were made of two seed trays (20 × 28 cm; Perma‐nest Plant Tray, Growers Supply Co., Inc.) placed 1 m apart. Each pair of arenas included two randomly assigned treatments: covered and uncovered. Covered arenas had an opaque shade cloth suspended 5–10 cm above the top edge of the tray, whereas uncovered arenas had no shade. Each arena contained 1.5 L of play sand with millet seed mixed into the sand. Each arena was prebaited with 6 g of millet (sand and seed accessible to foragers) for one night prior to the experimental run. At dusk (~20:00) of the night of the experimental run, we filtered out any remaining seeds and thoroughly mixed 4 g of fresh seeds into the sand in each arena. Because the only other major small mammal granivore at our field site, the eastern chipmunk (*Tamias striatus*), is strictly diurnal, having the arenas open only at night guaranteed that only white‐footed mice were foraging in the arenas.

We used general linear models to assess whether differences in GUDs between covered and uncovered seed trays can be explained by *Bb* infection of the mouse foragers. Models included trapping grid as covariate to better estimate the proportion of the variance that can be explained by *Bb* infection status alone.

### 2.5 Measuring predation on gypsy moths

In July 2015, after completion of the foraging assays described above, we affixed live gypsy moth pupae to burlap panels using beeswax. We deployed these on 80 evenly spaced trees per grid, with one pupa per tree at a height of 1.5 m. We obtained sterile, female, gypsy moth pupae from the USDA‐APHIS Otis Plant Protection Laboratory. Following Schauber et al. (2007), we checked the pupae daily for predation and signs of rodent visitation, including incisor marks in the beeswax, for a period of 7 days. To avoid affecting the native populations of gypsy moths, we destroyed all uneclosed pupae and any moths that eclosed after 1 week.

Rates of visitation and predation by infected and uninfected mice were compared by simple parametric tests. We compared the proportion of pupae that had been removed by the seventh day between grids receiving either true vaccine or sham vaccine. This seven‐day period reflects the typical duration of pupation of gypsy moths at our field sites (Jones et al., 1998).

### 2.6 Analytical methods

We used Kruskal–Wallis nonparametric tests to detect differences in continuous outcomes between the treatment and control groups. For multivariate analyses, we used generalized linear regressions with either a logit (for binary data) or Poisson (for count data) link. For counts of ticks on mice, which were overdispersed, we used a negative binomial regression.

All analyses were performed under the intention‐to‐treat scenario; that is, mice on grids assigned to a treatment category were considered treated whether or not they received the vaccination. All analyses were performed in the R statistical environment (R Core Team 2017). We present effect sizes (mean differences in the magnitude of response variables between treatment and control groups) along with the associated probabilities that the differences occurred by chance (*p*‐values).

## 3 RESULTS

**Figure 2:**
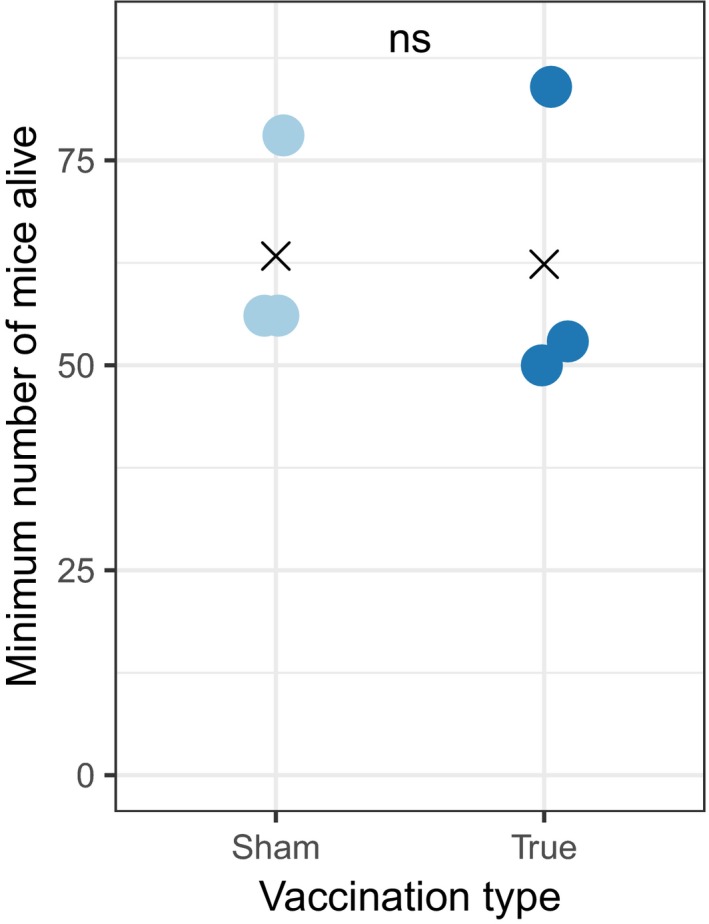
Density (measured as minimum number alive) of white‐footed mice (*Peromyscus leucopus*) did not differ between grids on which mice were treated with sham versus true vaccine. Kruskal–Wallis *p* = .37; × indicates the mean of the values. ns, not significant

In 2015, we vaccinated a total of 1,673 white‐footed mice, with 878 animals receiving the true vaccine and 795 receiving the sham vaccine. Of the mice temporarily transported to the laboratory to collect naturally feeding larval ticks, 53% produced at least one infected tick, regardless of vaccine treatment. Of those mice that received the sham vaccine, 62% produced at least one infected tick, while only 44% of mice that received the true vaccine did. Treatment with the true vaccine significantly reduced mouse reservoir competence; mice treated with the sham vaccine infected an average of 44% of feeding ticks, while mice treated with the true vaccine infected only 23% (Figure 1a; *p* < .001 from a Poisson regression with total number positive as the outcome variable and total number of ticks processed as an offset). By the time we began our experiments testing the effects of mice on predation of seeds and gypsy moth pupae, ~70% of mice on the grids had been treated with either the real or the sham vaccine (Figure 1b). Mice that had not been treated were young animals that weighed <10 g and were too small to receive vaccinations. There was no significant difference in mouse density on grids on which mice were treated with the true vaccine compared to those on which mice were treated with the sham vaccine (Figure 2; mean 63.33 vs. 62.33 [minimum number alive] Kruskal–Wallis *p* = .37).

**Figure 3:**
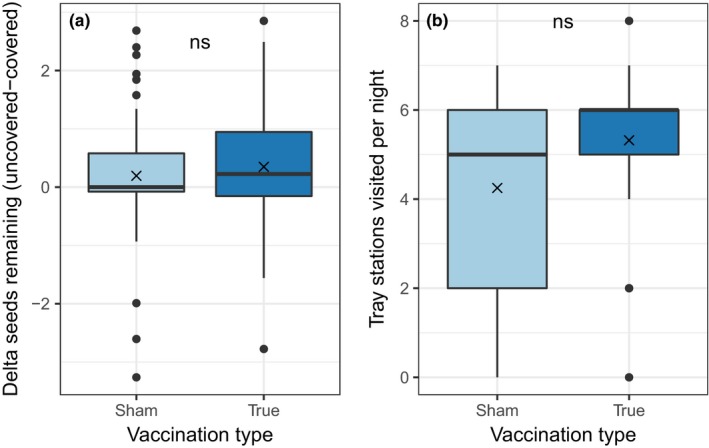
(a) Difference in weight of seeds remaining in uncovered versus covered trays when mice were treated with either sham or true vaccine. A bigger positive difference indicates greater sensitivity to predation risk. Horizontal lines within bars represent means of the differences between the covered and uncovered tray pairs on in each treatment. Only tray pairs for which at least one of the trays was visited by a mouse were included in this analysis. (b) Number of nights on which a tray station was visited on grids where mice were treated with either sham or true vaccine. × denotes the mean value. ns = not significant

Mice on all grids preferred covered foraging trays, which they apparently perceive as less risky. Overall, seed trays that were covered had ~15% less seed remaining than seed trays that were uncovered (covered: 1.8 g, uncovered: 2.1 g). The difference in seed remaining between the covered and uncovered tray in each pair was not significantly affected by vaccination treatment (Figure 3a; Kruskal–Wallis *p* = .251), indicating that vaccination type (true versus sham) did not affect the sensitivity of mice to predation risk. Seed trays on grids where mice received the true vaccine were 20% more likely to have been visited by foraging mice compared to seed trays on grids receiving the sham vaccine, although this difference was not statistically significant (Kruskal–Wallis *p* = .061). After controlling for site in a Poisson regression, we estimate there were 1.3 times as many visitations of seed trays on the true‐vaccinated grids, although the difference was also not statistically significant (*p* = .155).

**Figure 4:**
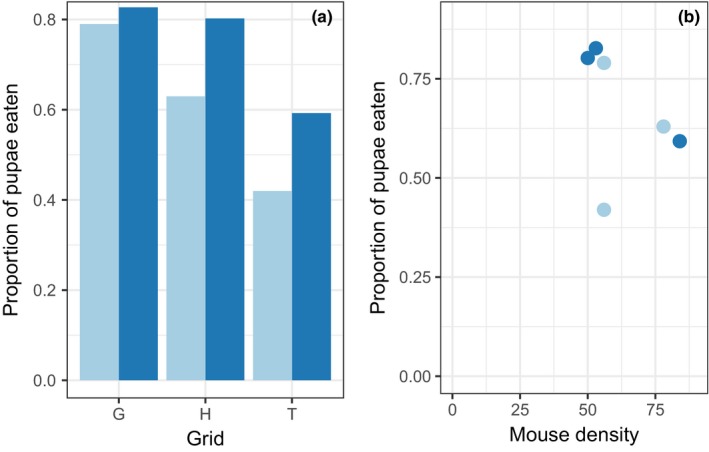
(a) Proportion of gypsy moth pupae eaten was consistently greater on grids occupied by mice treated with the true (dark blue) vaccine than on paired grids treated with the sham (light blue) vaccine, although the effect of vaccination treatment on the proportion of pupae eaten was not statistically significant (paired *t* = 2.82; *p* = .106). (b) Proportion of pupae eaten on each grid versus the minimum number of white‐footed mice known alive (MNA) on the grid at the time, with grids on which mice were treated with either sham (light blue) or true (dark blue) vaccine (Spearman rank correlation ρ = −0.69, *p* = .124)

The percentage of gypsy moth pupae eaten over 7 days ranged from a low of 42% to a high of 82% and was not a function of mouse density on the grids (Figure 4b). For all three grid pairs, the number of pupae eaten by mice (of a total of 81) was greater on grids receiving the true vaccine versus the paired grid on which mice were treated with the sham vaccine, but the difference between means was not statistically significant (Figure 4a; paired *t* = 2.82; *p* = .106).

**Figure 5:**
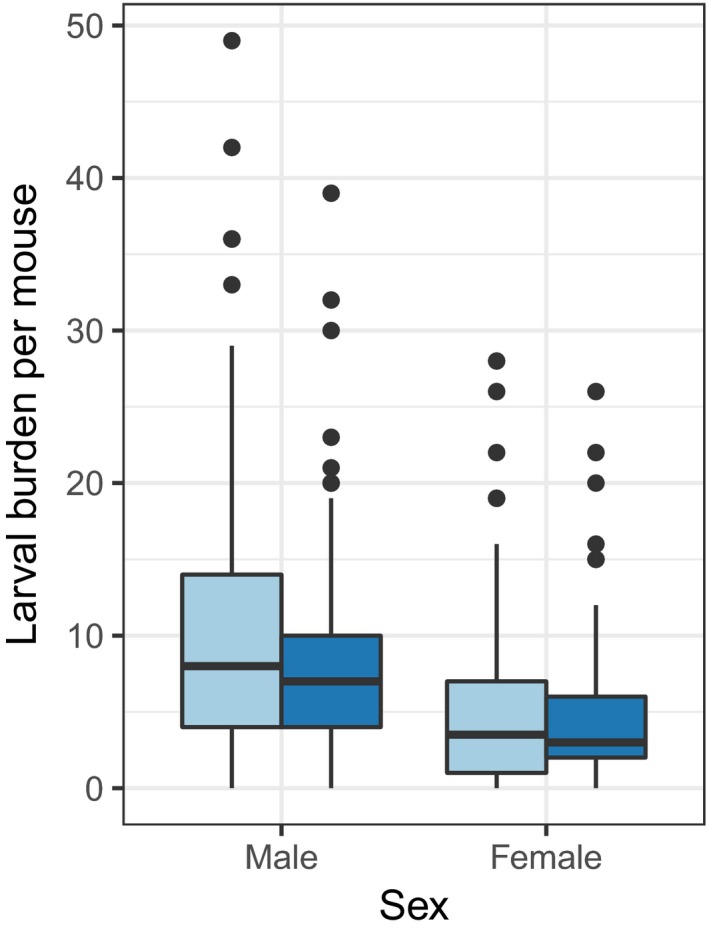
Both male and female mice on true‐vaccinated grids (dark blue) had lower larval tick burdens than mice on sham‐vaccinated grids (light blue) (*p* = .017, negative binomial regression). Horizontal lines within bars represent the mean larval burden for all mice of a given sex within that treatment category

The average mouse was infested with 7.4 ± 0.38 larval ticks on its head and ears. Male mice had almost twice as many ticks as female mice (males: 9.4 ± 0.6 standard error of the mean, females: 5.1 ± 0.4), a difference that was statistically significant (*p* < .001; Figure 5). Vaccinated mice had an average of 6.57 larval ticks, significantly fewer than the 8.23 larval ticks on control mice (*p* = .017, negative binomial regression). The difference was also significant after controlling for sex (*p* = .025); we found no evidence of an interaction between treatment and sex (*p* = .46).

## 4 DISCUSSION

The true anti‐*Bb* vaccine reduced the reservoir competence of mice, measured by their transmission of bacteria to ticks, by half. By vaccinating mice on large field plots (trapping grids), we created populations with lower (true vaccine) and higher (sham vaccine) average bacterial titers. We assessed whether mice on grids treated with the true vaccine differed from those treated with the sham vaccine in several direct and indirect measures of behavior relevant to forest interaction webs. The behavioral indicators we chose included: (1) risk aversion, measured by the tendency to forage more under covered than uncovered seed trays; (2) foraging activity on seeds, measured by the tendency to visit any seed tray; (3) foraging activity on gypsy moths, measured by attack rates on experimentally deployed moth pupae; and (4) regulation of tick burden, as measured by numbers of larval ticks parasitizing mice. If *Bb* is pathogenic to mice, a lower *Bb* titer should increase their overall health. Therefore, we expected true‐vaccinated mice to show indications of greater foraging and grooming activities compared to sham‐vaccinated mice. These behavioral parameters were estimated at the level of the population (i.e., trapping grid), resulting in a sample size of three pairs of true‐vaccinated and sham‐vaccinated grids, with each of the six grids maintaining a population of roughly 50–80 mice. Recognizing that our small sample of populations constrains statistical sensitivity, we report on effect sizes and consistency among grid pairs, as well as whether effects approached statistical significance at the traditional level of alpha = 0.05.

Mice treated with the true vaccine showed no increase in their sensitivity to predation risk, as would be indicated by differences in their preference for covered versus uncovered seed trays. Mice treated with the true vaccine were 25% more likely to visit seed trays than mice treated with the sham vaccine, although this difference was not statistically significant. Greater visitation rates would suggest that mice with lower bacterial titers ranged more widely or foraged more actively than their more heavily infected counterparts. Mice treated with the true vaccine appeared to be more likely to eat gypsy moth pupae than were mice treated with the sham vaccine, which also suggests greater foraging activity. Although this difference was observed across all the three grid pairs, it was not statistically significant. Finally, mice treated with the true vaccine hosted 20% fewer larval blacklegged ticks compared to mice treated with the sham vaccine, a statistically significant difference. This reduced tick burden suggests that mice with lower *B. burgdorferi* titers were more efficient at grooming ectoparasites, which can regulate larval tick burdens (Keesing et al., 2009). Together, the data suggest that true vaccination, and consequent reduction in *Bb* infection, appears to have at least subtle, but potentially important, effects on mouse behavior.

The true vaccine reduced the probability that mice would transmit *B. burgdorferi* to feeding ticks by half. However, it does not eliminate infection completely, in agreement with Tsao, Barbour, Luke, Fikrig, & Fish, 2001 and Richer, Brisson, Melo, Ostfeld, Zeidner, & Gomes‐Solecki, 2014. Consequently, our results should be interpreted as assessing the impact of reducing, rather than eliminating, infection of mice with this zoonotic pathogen. Moreover, the occurrence of uninfected mice in both vaccinated and sham‐vaccinated treatment plots would reduce the magnitude of differences between experimental and control treatments.

Prior studies of the effects of *B. burgdorferi* on mouse physiology and behavior have been mixed. For example, Moody, Terwilliger, Hansen, and Barthold (1994) found that young mice exhibited carditis and arthritis as a result of infection, while adult mice did not. Neither Hofmeister et al. (1999) nor Schwanz, Voordouw, et al. (2011) and Schwanz, Brisson, et al. (2011) detected changes in mouse activity as a result of infection with *B. burgdorferi*, but Burgess et al. (1990) found evidence of motor dysfunction in wild‐caught mice that seemed to be attributable to infection with *B. burgdorferi*.

The effects of infection with a zoonotic pathogen on a reservoir host can range from lethal to undetectable. For instance, infection with the plague bacterium *Yersinia pestis* is typically lethal to prairie dogs (genus *Cynomys*) (Eads & Biggins, 2015), causing local extinction of host populations (Cully, Johnson, Collinge, & Ray, 2010). On the other hand, *Y. pestis* only modestly reduces survival probability in some gerbillid hosts (Begon, 2006) and has variable impacts on survival of infected *Rattus rattus* (Tollenaere et al., 2010). Infection with hantaviruses rarely has detectable effects on health or survival of rodent hosts (Previtali et al., 2010; Tersago et al., 2008). Even when a zoonotic pathogen has only modest effects on host survival, it could potentially affect community dynamics if it influences host behavior in ways that might change trophic interactions.

The significant effect of vaccination in reducing tick burdens (Figure 5) suggests a potentially important effect of the pathogen on mouse behavior that could affect *Bb* transmission dynamics. We expected that a lower *Bb* titer could affect tick burdens if it either increased total ranging behavior (which would increase tick burdens) or increased grooming behavior (which would decrease tick burdens). Our results reject the former and support the latter hypothesis. Consequently, the potential exists for a positive feedback loop, whereby high *Bb* titers increase mouse encounters with ticks, which in turn should increase mouse infection with *Bb*. Whether the effect of *Bb* on tick burden was caused by sickness behavior of the host or by pathogen manipulation of host behavior is not apparent. Whether reduced *Bb* titer affects the other behaviors we assessed is more difficult to interpret. Although the magnitude of the increased visitation of seed trays seemed considerable (20%–30%), the differences were not statistically significant. Similarly, the increase in attack rates on gypsy moths on grids on which *Bb* titers were reduced, although observed across all three grid pairs, was not significant, challenging our ability to interpret these results.

Scientists have begun discussing editing the genome of *P. leucopus* to make them unable to serve as reservoirs for *Bb* (Pennisi, Elizabeth. “U.S. researchers call for greater oversight of powerful genetic technology.” Science. July 17, 2014.) This proposal, while still in planning stages, raises important questions about potential ramifications of such a manipulation. Because of the status of white‐footed mice as ecological hubs, investigating the ecological consequences of *Bb*‐free mice is even more pressing. Current explorations of the use of genetic technology to create populations of white‐footed mice that are refractory to *Bb* infection must include information on the potential for unintended consequences of reduced infection. We have explored a small set of these potential consequences and found evidence of only minor impacts on foraging behavior but potentially important effects on tick burdens that we interpret as a consequence of altered host grooming behavior. Prior research has indicated that many of the effects of white‐footed mice on prey, such as gypsy moths and songbird nests, are a consequence of incidental encounters with these highly generalist foragers (Schauber et al., 2007; Schmidt et al., 2001). Further studies will be necessary to ask whether reduced or eliminated *Bb* infection affects infection with other zoonotic pathogens and other parasites, interactions with predators on reservoir hosts, and additional behavioral features, including long‐distance dispersal and habitat selection.

We chose to conduct an experimental test of the effects of *Bb* infection on host behavior because of severe shortcomings inherent in correlational studies. Causality can be difficult or impossible to establish in studies using nonexperimental comparisons of the behavior of infected versus uninfected hosts. For instance, behavioral differences between hosts that vary naturally in their infection status could be the cause of their varying infection status rather than a consequence. Our use of a sham vaccine was important in accounting for the potential for immunochallenge itself to affect host behavior (Schwanz et al., 2012). Nevertheless, our experimental manipulation of *Bb* infection with a real and sham vaccine did not create uninfected and infected categories, as we had intended, instead only reducing pathogen titers, as measured by reservoir competence. Further studies in which infection is eliminated in reservoir populations are warranted.

## CONFLICT OF INTEREST

None declared.

## AUTHOR CONTRIBUTIONS

RSO, DB, and FK conceived of the project; RSO, DB, MZL, and FK designed the experiments; KO led the field efforts; JD and DB led the laboratory efforts; FK, MZL, and DB analyzed the results; RSO and FK wrote the first draft of the manuscript; and all authors edited the manuscript.
